# FPGA-Implemented Fractal Decoder with Forward Error Correction in Short-Reach Optical Interconnects

**DOI:** 10.3390/e24010122

**Published:** 2022-01-13

**Authors:** Svitlana Matsenko, Oleksiy Borysenko, Sandis Spolitis, Aleksejs Udalcovs, Lilita Gegere, Aleksandr Krotov, Oskars Ozolins, Vjaceslavs Bobrovs

**Affiliations:** 1 Communication Technologies Research Center, Riga Technical University, 1048 Riga, Latvia; Sandis.Spolitis@rtu.lv; 2 Department of Electronics and Computer Technology, Sumy State University, 40007 Sumy, Ukraine; Oleksiy.Borysenko@ekt.sumdu.edu.ua; 3 Institute of Telecommunications, Riga Technical University, 1048 Riga, Latvia; Lilita.Gegere@rtu.lv (L.G.); Aleksandr.Krotov@rtu.lv (A.K.); Oskars.Ozolins@ri.se (O.O.); Vjaceslavs.Bobrovs@rtu.lv (V.B.); 4 Networks Unit, RISE Research Institutes of Sweden, Kista, 164 40 Stockholm, Sweden; Aleksejs.Udalcovs@gmail.com; 5 Applied Physics Department, KTH Royal Institute of Technology, 106 91 Stockholm, Sweden

**Keywords:** coded modulation, error-correcting codes, error-detecting codes, indivisible codes, fractal decoder, short-reach optical interconnects

## Abstract

Forward error correction (FEC) codes combined with high-order modulator formats, i.e., coded modulation (CM), are essential in optical communication networks to achieve highly efficient and reliable communication. The task of providing additional error control in the design of CM systems with high-performance requirements remains urgent. As an additional control of CM systems, we propose to use indivisible error detection codes based on a positional number system. In this work, we evaluated the indivisible code using the average probability method (APM) for the binary symmetric channel (BSC), which has the simplicity, versatility and reliability of the estimate, which is close to reality. The APM allows for evaluation and compares indivisible codes according to parameters of correct transmission, and detectable and undetectable errors. Indivisible codes allow for the end-to-end (E2E) control of the transmission and processing of information in digital systems and design devices with a regular structure and high speed. This study researched a fractal decoder device for additional error control, implemented in field-programmable gate array (FPGA) software with FEC for short-reach optical interconnects with multilevel pulse amplitude (PAM-M) modulated with Gray code mapping. Indivisible codes with natural redundancy require far fewer hardware costs to develop and implement encoding and decoding devices with a sufficiently high error detection efficiency. We achieved a reduction in hardware costs for a fractal decoder by using the fractal property of the indivisible code from 10% to 30% for different *n* while receiving the reciprocal of the golden ratio.

## 1. Introduction

With the development of the 5G network architecture, 4K/8K video streaming, the Internet of Things (IoT), cloud computing, and other modern technologies, the task of improving the reliability of data transmission remains urgent [1]. With an increase in the volume of the transmitted information and growing traffic, a strong candidate is PAM-M since it possesses cost-effectiveness, spectral efficiency, energy efficiency, and simplicity [2,3,4,5,6]. Although it is studied for short-reach networks, PAM-M format modulation was considered for C-band mobile and interdata centre networks.

Optical communication systems need to maintain highly efficient and highly reliable communications using digital signal processing (DSP), forward error correction (FEC), and modern coherent technologies via techniques such as pulse shaping. Using high spectral efficiency (SE) modulation formats and powerful FEC techniques is one way to address the Internet’s exponential traffic growth and improve transmission reach. In theory of codes, there are a few types of FEC codes, such as hard-input hard-output (HIHO) and soft-input soft-output (SISO) decoders. HIHO decoding uses hard values, in which the output is quantized only to two levels. In contrast, SISO decoding uses soft values, where confidence information for the decision is not just one or zero decisions. This “soft information” is often represented using logarithmic likelihood ratios (LLRs) that operate as the input of soft-decision FEC decoders [7,8,9,10].

When post-FEC bit error rate (BER) is concerned, a minimum of 10^−12^ or preferably 10^−15^ is generally required [11]. HD-FEC codes that meet system needs of 100 Gb/s and beyond typically have a more negligible overhead, and do not require costly A/D converters for their implementation. From this type of code, the best net coding gain (NCG) at 10^−15^ post-FEC BER is provided by concatenated interleaved Bose–Chaudhuri–Hocquenghem (CI)-(BCH) 4 with a 20% overhead (OH) [12,13] and Turbo Product code with shortened BCH component codes [14], whereas CI-BCH 4 with 7% (OH), Swizzle [15] and Staircase codes [16] achieves the most outstanding performance for a standard (7%) OH. From the class of SD-FEC codes, the spatially coupled type low-density parity-check (LDPC) code [17] exhibits the highest NCG at a 10^−15^ post-FEC BER, with OH is at 5% higher than that of the other described FEC codes. LDPC-CCs [18] and ViaSat’s TPCs [19,20] are the two implemented schemes with the greatest correcting performance with a 20% OH at a post-FEC BER of 10^−15^. Some of the other proposed LDPC-based codes also achieve similar or even better performance (e.g., large-girth LDPC [21,22] or nonbinary QC-LDPCs [22,23]).

Positional number systems, on the basis of which indivisible codes are generated, such as noninteger position number systems based on the golden ratio, Fibonacci [24,25,26,27,28], binomial, and permutation codes, were researched in [29,30], or quaternary imaginary number systems [31]. The Fibonacci code is an error detection code capable of detecting errors in both transfers of information and processing, i.e., has an end-to-end (E2E) control property. This code is most effective in asymmetric channels for processing and transmitting information in which the errors’ type is 0→1. This asymmetry is compensated for by using FEC codes with artificial redundancy, thereby obtaining a cumulative effect of improved interference resistance [24,25,26,27,28]. The Fibonacci code is effectively used for computer arithmetic and developing digital devices on their basis. A new method for representing images using a nonstandard positional number system was developed and considered [32]. The residue number system (RNS) arithmetic is effectively used in design technologies and new applications in cryptography, machine learning, and deep convolutional neural networks, Internet of Things (IoT) devices, postquantum algorithms and circuits, embedded processor design, and other related emergent topics [33,34,35,36].

The main criterion for any error control codes (ECC) is the use of redundant information contained in the encoded words, which is typically artificially introduced into them. Another type of redundant coding uses natural redundancy in the codewords. The application of natural redundancy codes requires far less hardware to synthesize encoding and decoding devices, and uses more straightforward methods to accomplish these tasks. Indivisible codes are practical to use for constructing coding and decoding devices. An end-to-end (E2E) control is possible in them, both transmitting information and processing, which can considerably improve their efficiency. The positive effect is achieved due to the use of indivisible codes to control digital devices that process information, and for communication channels. It reduces hardware expenses required for the work of a network, and increases performance speed and reliability. In addition, the application of hybrid types of ECC in optical communication systems using natural redundancy can be significantly enhanced by using codes with artificial redundancy. In this way, using relatively fewer complex algorithms in encoding and decoding devices for hardware implementation can achieve more effective indicators to protect transmitted and processed data.

E2E data control enables both transmission and digital devices, which significantly improves the efficiency of information systems. Codes involving artificial redundancy are designed only for error control tasks in either communication channels or information processing systems and do not allow for E2E data control. This creates effectively evaluating indivisible error detection codes with natural redundancy for their further application in digital devices and systems. As an additional control, indivisible codes can be used in conjunction with FEC codes, making it possible to simplify implementation methods of encoding and decoding devices with significant error detection properties.

In this paper, we investigate a fractal decoder, which was implemented in FPGA using Intel Quartus Prime software. The device was investigated for a 56 and 35 Gbaud PAM- M (M = 4, 8) modulation with Gray code mapping using wavelength division multiplexed (WDM) optical interconnect model with a standard single-mode fibre (SSMF) and erbium-doped fibre amplifier (EDFA). We investigate the combination of an indivisible natural redundancy error detection code with an artificial redundancy FEC based on the interleaved BCH + LDPC and RS + LDPC FEC. We also evaluate the indivisible error detecting code using the average probability method (APM), which demonstrated that the simplicity, versatility, and reliability of estimation is close to reality. This shows that it is possible to reduce the saving hardware costs of the fractal decoder by using the fractal property of indivisible code. An increase in the savings in hardware costs occurs with an increase in the code length of the indivisible code, which is limited to the reciprocal of the golden ratio. With fractal decoding, the signal delays twice as much as the standard method of constructing line Fibonacci decoders.

The paper is structured as follows: Section 2 presents the theoretical aspects and estimation of the indivisible code based on the APM for a binary symmetric channel (BSC). Section 3 analyses the 56 and 35 Gbaud wavelength division multiplexed (WDM) short-reach optical interconnects for PAM-M (M = 4, 8) modulation with Gray code mapping with SSMF and EDFA. EDFA is used for signal amplification and to investigate the code performance in the presence of ASE noise. The simulation results of the WDM optical interconnect model with interleaved BCH + LDPC, and RS + LDPC with the fractal decoder device are also presented in Section 3. Section 4 investigates the fractal decoder device for the indivisible error-detecting code implemented in FPGA software. Lastly, we conclude this paper in Section 5.

## 2. Theoretical Aspects and Estimation of Indivisible Code Based on APM

### 2.1. Theoretical Aspects of Indivisible Code

In this paper, we use the Fibonacci indivisible error-detection code. The Fibonacci number system makes it possible to generate the Fibonacci code, consisting of Fibonacci numbers whose weights are a sequence of numbers 1, 1, 2, 3, 5, 8, …, *F_n_*.

Equation (1) defines a sequence of numbers in an indivisible code [37,38,39]:

(1) $$F_{n}=F_{n-1}+F_{n-2}.$$

It follows from Equation (1) that each subsequent element of the Fibonacci series is equal to the sum of its two preceding elements. The quantitative value of the Fibonacci numbers is set by a numbering function whose weights are the Fibonacci numbers represented as Equation (2) [25,26,27,28]:

(2) $$N=a_{n}F_{n}+a_{n-1}F_{n-1}+\ldots +a_{i}F_{i}+\ldots +a_{1}F_{1}$$

where *a*_*i*_ ∈ {0, 1} is the binary digit of the *i*-th bit in the positional representation of a number; *n* is the length of the code; *F*_*i*_ is the weight of the *i*-th bit, which is equal to the *i*-th Fibonacci numbers. The abbreviated form of Equation (1) is shown as Equation (3) [25,26,27,28]:

(3) $$N_{a}=a_{n}a_{n-1},\ldots ,a_{i},\ldots ,a_{1}.$$

Table 1 shows the indivisible code for *n* = 8 and numbers *N* = 25, 54, 33, with bit weights 1, 2, 3, 5, 8, 13, 21, 34.

The range of Fibonacci numbers is determined from Equation (4) [25,26,27,28]:

(4) $$P=F_{n}+F_{n-1}$$

where *F_n_* is the weight of the *n*-th bit of the Fibonacci numbers; *F_n_*_−1_ is the weight of the *n*−1 bits of the Fibonacci numbers.

Using two binary numbers, the range of Fibonacci numbers equals *P*_2_ = 1 + 1 = 2, for *P*_3_ = 2 + 3 = 5, for *P*_4_ = 3 + 5 = 8. This code prohibits having two unities side by side, which is a sign of an error. If there are three neighbouring unities in the code combination, the middle bits can be corrected by inverting it to zero, resulting in a correction in the error-detecting code. Thus, the indivisible code can detect errors and correct some, transforming this code into an FEC code.

The fractal structure of the indivisible code with *n* = 5 is shown in Table 2.

The indivisible code possesses the property of self-similarity consisting of fractals with several ranks. Equation (1) forms the fractal of the first rank, from which multifractals of the second, third, etc. are constructed ranks. Table 2 shows that the fractals of the first rank are the first five bits with 0 in the MSB, and the last five bits with 1 in the MSB. Bits 0 or 1 in the MSB are identifier fractals 1 and 2. Thus, they are enough to decode one of the fractals and save hardware costs. The line decoder decodes the nonfractal part of the code.

### 2.2. Estimation of Indivisible Code Based on Average Probability Method (APM)

We applied the APM for the BSC to estimate the indivisible code. The APM determines the probabilities of the transitions of code combinations of an indivisible code into proper, allowed, and prohibited classes. The APM possesses simplicity, versatility, and reliability of estimation, close to reality [40].

The probability of the proper transition of the indivisible code is represented by Equation (5):

(5) $$C=\sum_{i=1}^{M}P_{i}p_{i}^{i}$$

where *P_i_* is the probability that an information source generates the *i*-th code combination; $p_{i}^{i}$ is the probability that the *i*-th code combination is properly transferred into the *i*-th code combination.

The probability of undetectable erroneous transitions of the indivisible code is represented by Equation (6):

(6) $$V=\sum_{i=1}^{M}P_{i}p_{i}^{u},$$

where $p_{i}^{u}$ is the probability that the *i*-th code combination is erroneously transferred into the class of code combinations that is not detected.

The probability of an erroneous transition of the indivisible code is represented by Equation (7):

(7) $$p_{i}^{u}=\sum_{j=1,j\neq i}^{M}p_{i,j}^{u}$$

where $p_{i,j}^{u}$ is the probability that the *i*-th code combination is erroneously transferred into the *j*-th allowable code combination.

The probability of the detected error is determined from Equation (8):

(8) $$Z=\sum_{j=1}^{M}P_{i}p_{i}^{d}$$

where $p_{i}^{d}$ is the probability that the *i*-th code combination is erroneously transferred into the *j*-th allowable code combination, and represented by Equation (9):

(9) $$p_{i}^{d}=\sum_{j=M+1}^{N}p_{i,j}^{d}\;$$

where $p_{i,j}^{d}$ is the probability that the *i*-th code combination is erroneously transferred into the *j*-th prohibited combination.

**Figure 1:**
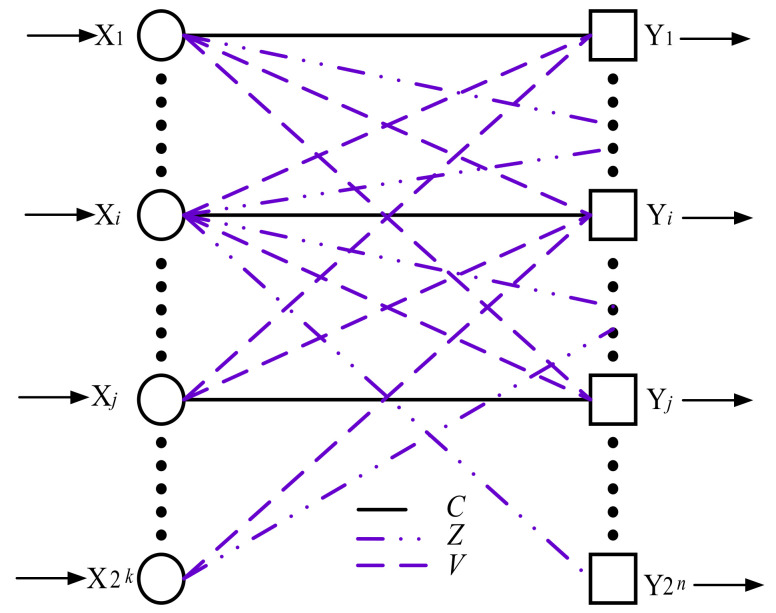
Possible transformations of code combinations of indivisible code into classes *C*, *V*, *Z*.

Figure 1 shows the possible transformations of $2^{k}$ code combinations of indivisible code into classes *C*, *V*, *Z*.

The input data for the APM are the probabilities of the transition of 0 to 0 (*p*_00_) and 1 to 1 (*p*_11_). The probability of an erroneous transition of 0 to 1 and 1 to 0 is determined by *p*_01_ = 1 − *p*_00_, *p*_10_ = 1 − *p*_11_.

**Figure 2:**
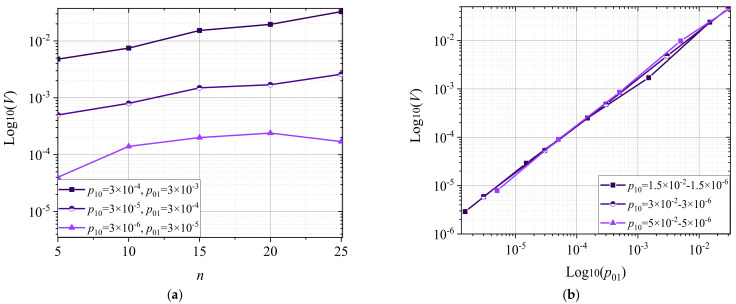
Probability of proper data transmission of indivisible code (**a**) Log_10_(*C*) from *n* for *p*_10_ = 3 × 10^−4^; 3 × 10^−5^; 3 × 10^−6^, and *p*_01_ = 3 × 10^−3^; 3 × 10^−4^; 3 × 10^−5^; (**b**) Log_10_(*C*) from Log_10_(*p*_10_) for *p*_10_ = 1.5 × 10^−3^–1.5 × 10^−7^; 3 × 10^−3^–3 × 10^−7^; 5 × 10^−3^–5 × 10^−7^.

Figure 2 shows the probability of the proper data transmission of the indivisible code: (a) Log_10_(*C*) from *n*, for example, for *p*_10_ = 3 × 10^−4^; 3 × 10^−5^; 3 × 10^−6^, and *p*_01_ = 3 × 10^−3^; 3 × 10^−4^; 3 × 10^−5^; (b) Log_10_(*C*) from Log_10_(*p*_10_) for *p*_10_ = 1.5 × 10^−3^–1.5 × 10^−7^; 3 × 10^−3^–3 × 10^−7^; 5 × 10^−3^–5 × 10^−7^, at *n* = 6.

Figure 2a shows that, with an increase in *n*, the number of bits in which an error of the indivisible code is possible increases. The probability of correct transmission decreases with increasing *n*, but in some cases, it reaches 99.9%. Figure 2b shows that, with the probability value (*p*_10_) decreasing, the likelihood of correct data transmission increases at *n* = 6.

**Figure 3:**
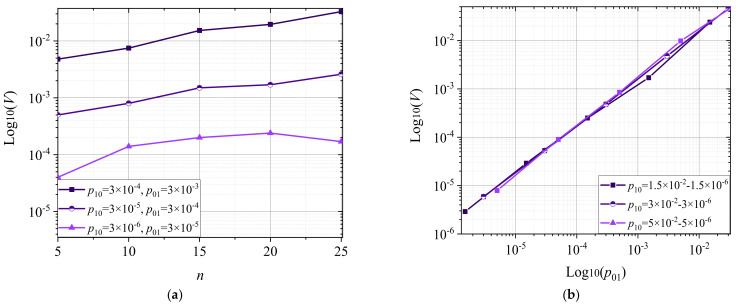
Probability of an undetectable error of the indivisible code (**a**) Log_10_(*V*) from *n* for *p*_10_ = 3 × 10^−4^; 3 × 10^−5^; 3 × 10^−6^, and *p*_01_ = 3 × 10^−3^; 3 × 10^−4^; 3 × 10^−5^; (**b**) Log_10_(*V*) from Log_10_(*p*_01_) for *p*_01_ = 1.5 × 10^−3^–1.5 × 10^−6^; 3 × 10^−3^–3 × 10^−6^; 5 × 10^−3^–5 × 10^−6^.

Figure 3 shows the probability of an undetectable error of the indivisible code: (a) Log_10_(*V*) from *n* for *p*_10_ = 3 × 10^−4^; 3 × 10^−5^; 3 × 10^−6^, and *p*_01_ = 3 × 10^−3^; 3 × 10^−4^; 3 × 10^−5^; (b) Log_10_(*V*) from Log_10_(*p*_01_) for *p*_01_ = 1.5 × 10^−3^–1.5 × 10^−6^; 3 × 10^−3^–3 × 10^−6^; 5 × 10^−3^–5 × 10^−6^, at *n* = 6.

Figure 3a shows that the probability of an undetectable error of the indivisible code increases with *n*. Figure 3b shows that, with a decrease in the probability value (*p*_01_), the probability of an undetectable error *V* decreases at *n* = 6.

**Figure 4:**
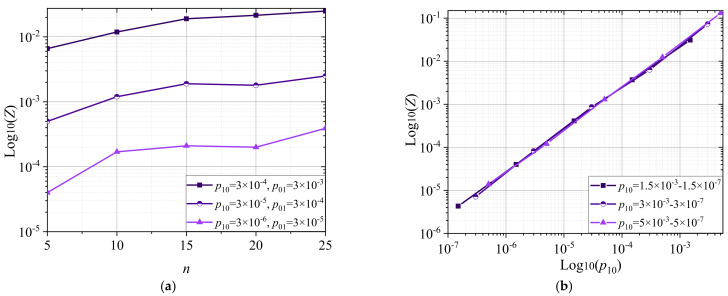
Probability of an error detected in the indivisible code. (**a**) Log_10_(*Z*) from *n* for *p*_10_ = 3 × 10^−4^; 3 × 10^−5^; 3 × 10^−6^, and *p*_01_ = 3 × 10^−3^; 3 × 10^−4^; 3 × 10^−5^; (**b**) Log_10_(*Z*) from Log_10_(*p*_01_) for *p*_01_ = 1.5 × 10^−3^–1.5 × 10^−7^; 3 × 10^−3^–3 × 10^−7^; 5 × 10^−3^–5 × 10^−7^.

Figure 4 shows the probability of a detected error of the indivisible code: (a) Log_10_(*Z*) from *n* for *p*_10_ = 3 × 10^−4^; 3 × 10^−5^; 3 × 10^−6^, and *p*_01_ = 3 × 10^−3^; 3 × 10^−4^; 3 × 10^−5^; (b) Log_10_(*Z*) from Log_10_(*p*_10_) for *p*_10_ = 1.5 × 10^−3^–1.5 × 10^−6^; 3 × 10^−3^–3 × 10^−6^; 5 × 10^−3^–5 × 10^−6^, at *n* = 6.

Figure 4a shows that the probability of a detected error in the indivisible code increases with *n*. Figure 4b shows that, with a decrease in the probability value (*p*_10_), the probability of the detected error decreases at *n* = 6. The APM allows for the evaluation of codes with any code distance, starting from code distance *d* = 1. The APM makes it possible to compare and estimate error probability, in both the processing and transmission of indivisible codes.

## 3. System Model, Achievable Rates and Numerical Analysis of an Optical System

### 3.1. System Model and Achievable Rates

**Figure 5:**
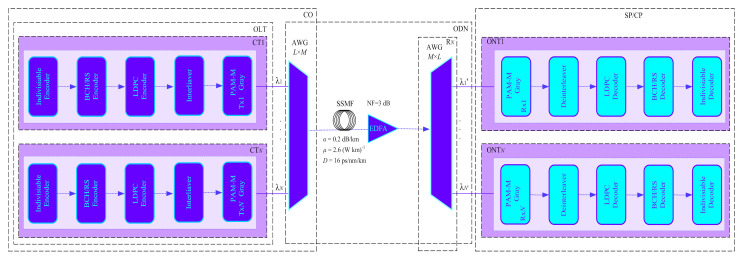
WDM optical interconnect model.

Figure 5 shows a five-channel WDM optical interconnect model that integrates concatenated FEC based on BCH + LDPC, RS + LDPC FEC with an interleaver or deinterleaver, and the indivisible code with the fractal decoder.

The architecture of the 5 WDM-channel PAM-M with Gray code mapping and simple pulse-shaping comprises a pseudorandom binary sequence (PRBS) generator; indivisible encoders; interleaved BCH + LDPC, RS + LDPC FEC encoders; optical transmitters (Tx_1_–Tx*_N_*), which consist of central office (CO); optical distribution network (ODN); subscriber premises/customer premises (SP/CP); optical line terminal (OLT); channel terminals (CT_1_–CT*_N_*), arrayed waveguide grating (AWGs) *N* × *M* multiplexers/*M* × *N* demultiplexers; single-mode fibre (SSMF) and EDFA amplifier with 3dB noise figure; remote node (R*_N_*); optical network terminals (ONT_1_–ONT*_N_*), which comprises the receivers (Rx_1_–RX*_N_*), interleaved BCH + LDPC, RS + LDPC FEC, and indivisible decoders.

The 5-channel WDM optical interconnect model with PAM-M modulation format was chosen as the primary system parameters: the channel spacing was chosen to be equal to 100 GHz according to the ITU-T G.694.1 recommendation [41], the central frequencies were set to 193.1 THz (optical *C*-band), and the data rate per channel was 56 and 35 Gbaud for PAM-M (*M* = 4, 8). The optical line terminal (OLT) comprises *N-*channel terminals (CT), comprising *N*-optical transmitters (OT), where *N* is the transmitter number. The CT*_N_* block includes the continuous wave (CW) lasers and Mach–Zehnder modulators (MZM) with a 30 dB extinction ratio, and CW lasers with an optical output power of +11 dBm. In the AWGs, the following parameters are used according to the commercial datasheet: an operation range of 193–193.5 GHz, a Gaussian passband, a 3 dB bandwidth of 75 GHz, and an insertion loss of 3 dB per channel.

Table 3 shows the simulation setup parameters for the 5 WDM optical interconnect.

The data are transmitted through a standard ITU-T G.652 SSMF span from OLT block with attenuation coefficient, 0.2 dB/km; dispersion slope, 0.08 ps/nm^2^; dispersion coefficient, 16 ps/(nm × km); and nonlinear index, 2.6 × 10^−20^ m^2^/W at reference wavelength 1550 nm [42]. The SP/CP includes *N* optical network terminals (ONTs), which consist of PIN receivers with thermal noise of 7.0 × 10^−12^ A/Hz^1/2^, dark current 5.0 × 10^−8^ A, 4-pole order low-pass Bessel electrical filter with 18.75 GHz frequency band, deinterleaved BCH + LDPC; RS + LDPC FEC, and indivisible fractal decoders.

### 3.2. Numerical Analysis of an Optical System

The WDM optical interconnect model used concatenated codes, for instance, the indivisible error-detection code, interleaved BCH + LDPC (with shortened BCH codes), and RS + LDPC FEC. We used the fractal decoder for indivisible code, Berlekamp–Massey algorithm (BMA) for BCH and RS, and belief propagation algorithm (BPA) for LDPC FEC.

The RS (15, 5) FEC code used 200% overhead (OH), and code rate *R*_c_ = 0.33. We considered the LDPC FEC code from the digital video broadcasting satellite second-generation standard with code rate *R*_c_ = 0.75 with 33.3% overhead (OH), block length of *n* = 64,800 bits, and 50 decoding iterations [43].

**Figure 6:**
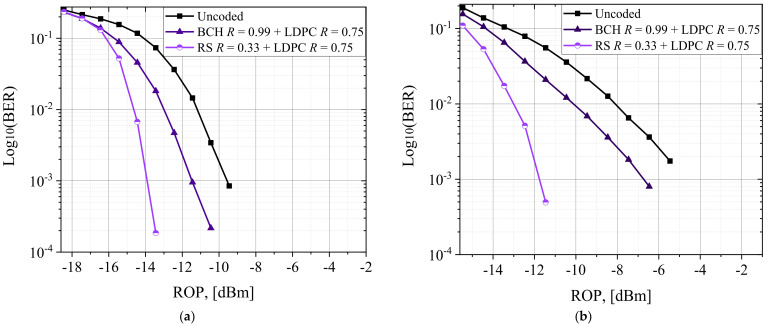
Post-FEC BER vs. ROP with 56 and 35 Gbaud B2B and 1000 m SSMF for (**a**) PAM-4 modulation format with interleaved BCH + LDPC and RS + LDPC FEC, and (**b**) PAM-8 modulation format with interleaved BCH + LDPC and RS + LDPC FEC.

**Figure 7:**
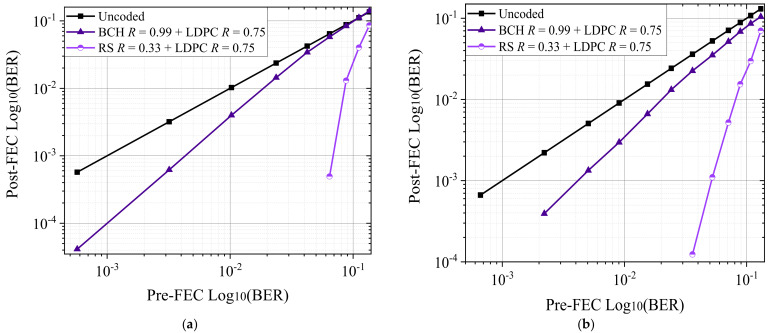
Post-FEC BER vs. pre-FEC BER with 56 and 35 Gbaud PAM-M (M = 4, 8) modulation formats with OSNR and EDFA for (**a**) PAM-4 modulation format with interleaved BCH + LDPC and RS + LDPC FEC, and (**b**) PAM-8 modulation format with LDPC, interleaved BCH + LDPC and RS + LDPC FEC.

We investigated the received optical power (ROP) and optical signal-to-noise ratio (OSNR) with concatenated FEC codes for the particular bit error rate (BER) for the WDM optical interconnect model. Figure 6a,b show the BER value change with the ROP of the central WDM channel. Transmission distances 1000 m for PAM-4 (56 Gbaud) and PAM-8 (35 Gbaud) modulation formats with interleaved BCH + LDPC and RS + LDPC FEC. Figure 7 shows the post-FEC BER vs. pre-FEC BER performance of central WDM channel for PAM-4 (56 Gbaud) and PAM-8 (35 Gbaud) modulated with an optical signal-to-noise ratio (OSNR) and EDFA for (a) PAM-4 modulation format with interleaved BCH + LDPC, and RS + LDPC FEC, (b) PAM-8 modulation format with interleaved BCH + LDPC and RS + LDPC FEC.

Error statistics and post-FEC simulations use the Monte Carlo (MC) BER measurement up to 10^−5^, using the standard [11] expected BER up to 10^−15^. Compared to the BCH + LDPC FEC, the RS + LDPC FEC coded system, of which the correction symbol errors are in each codeword, have the best post-FEC performance and the highest OH for simulation WDM optical interconnect model. Post-FEC BER values for interleaved BCH + LDPC and RS + LDPC, which are shown in Figure 6a,b at an ROP, were −18 and −10 dBm for PAM-4, and −15 and −6 dBm for PAM-8; at a higher ROP, post-FEC BER values were zero. The post-FEC BER for interleaved BCH + LDPC and RS + LDPC codes in Figure 7a,b were achieved at an OSNR within the 28–42 dBm and 36–49 dBm for PAM-4 and PAM-8, respectively.

## 4. Design of the Fractal Decoder Device

**Figure 8:**
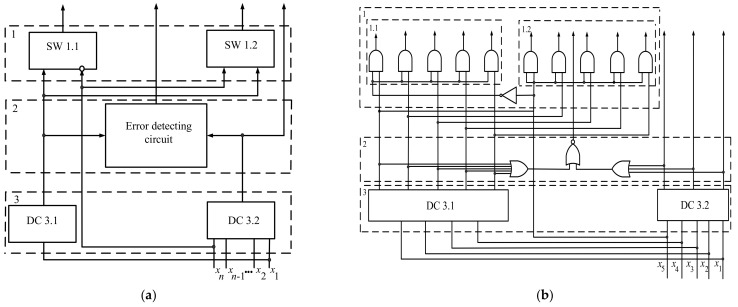
(**a**) Fractal decoder block diagram; (**b**) fractal decoder circuit diagram.

Figure 8a,b show the fractal decoder device’s block diagram and circuit diagram. The fractal decoder device includes a block of switching devices 1 (SW 1.1 and SW 1.2), block of the error detection circuit 2, and block of line decoders 3, comprising a decoder 3.1 (DC 3.1) with *n* − 1 input, forming the fractal part of the indivisible code with (*n* − 1)*F_n_*_−1_ inputs, and decoder 3.2 (DC 3.2) with *n* inputs, forming a nonfractal part of the indivisible code with *n*(*F_n_* − *F_n_*_−1_) = *n* ((*F_n_*_−1_ + *F_n_*_−2_) − *F_n_*_−1_) = *n × F_n_*_−2_ inputs. The switching devices (SW 1.1, SW 1.2) of block 1, connected to the DC 3.1 and DC 3.2, performed the commutative switching function of the DC 3.1 and DC 3.2 for the MSB or LSB on the basis of a value of 0 or 1. The MSB transmits to the inverter control input of SW 1.1, and at the same time to the direct control input of SW 1.2. If the signal is 0, then SW 1.1 is turned on; if it is 1, then SW 1.2. Accordingly, the signal from the output of DC 3.1 appeared at one of the outputs of SW 1.1 or SW 1.2. The total number of outputs of SW 1.1 and SW 1.2 of block 1 equalled 2 × *F_n_*_−1_.

Decoding the essence was as follows: 4 LSB of fractal 1 repeated in 4 LSB of fractal 2 in Table 2. The difference between these fractals lay in the content of zeros or units in the MSB. Thus, to decode code fractals, it is enough to decode one of them [15,16,17]. The nonfractal part of the indivisible code decodes with the use line decoder.

For example, code combinations of the indivisible code shown in Table 2 transmit to inputs DC 3.1 and DC 3.2. Decoder DC 3.1 decodes fractal 1 corresponding to code combinations 0–4 or 8–12. Decoder DC 3.2 interprets fractal 2, corresponding to code combinations 5–7. Depending on the MSB of the code combination of fractals 1 or 2, one of the elements of switching devices SW 1.1 or SW 1.2 is triggered. If, as a result of decoding fractal or nonfractal parts of the indivisible code, an error occurs, characterized by the appearance of two or more units that are next to each other in the code combinations, at the output of the error detection circuit, a signal of 1 is indicated.

The reduced hardware costs of the fractal decoder compared to the line decoder are due to using one fractal (fractal 1 or 2) in DC 3.1 to decode the code combinations of both fractals. However, instead of the excluded constituent, the fractal decoder uses the switching devices with 2-input OR elements, requiring much lower hardware costs than those of implementing components of the additional fractal part. Due to this, hardware costs are reduced, and this is more remarkable, the more significant the n of the indivisible code is.

Decoders DC 3.1 and DC 3.2 are the line decoders. However, opportunities became available to develop more economical decoder DC 3.1 by analogy with the fractal construction using the same structure as that of the DC 3.2 decoder. More economical decoders DC 3.1 can be implemented according to multifractal construction until the saved hardware costs are profitable. In this case, each subsequent DC 3.1 decoder for its synthesis uses the multifractal of a higher rank—third, fourth, etc.

For example, to implement the fractal decoder for 21 outputs on the basis of a fractal decoder with 13 outputs, the number 21 is represented as fractal equality of the first rank 21 = ((8 + 5) + 8), which indicates introducing an additional switching device for 16 outputs. Then, depending on the MSB of decoder 3.2, which includes eight inputs, they are triggered to one of the switching devices consisting of eight 2-input OR elements of the second stage with 16 outputs; 5 outputs in this case remain without switching. As a result, any of the 21st indivisible code combinations are decoded. In designing such multistage decoders, only multifractals of the first and second ranks are used that are then transformed, with the advent of switching devices, into fractals of second and third ranks, third and fourth, and so on, an unlimited number of ranks, thus constructing the multistage decoders.

The method of fractal decoding is as follows:

1. In a set of the indivisible code *f* = *x*_1_, *x*_2_, …, *x_j_*, …, *x_n_*, fractal parts are found that are distinguished by the presence of 0 or 1 in the MSB.
2. The fractal part of the indivisible codes decodes by the line decoder (D 3.1).
3. Depending on the signal of the MSB (0 or 1), the first (SW 1.1) or second switch (SW 1.2) is triggered, wherein its outputs correspond to the numbers of the first or second fractals.
4. The codes are not included in the fractal part decoding by the line decoder (D 3.2).
5. If an erroneous combination is received at the inputs of decoders DC 3.1 and DC 3.2, in which *x_j_* × *x*_−-1_ = 1, *j* = 1, 2, …, *n*, an error signal is detected.

The primary indicator in developing a fractal decoder device is saving hardware costs, which are reduced. As a measure for calculating hardware costs, we counted the number of inputs of logical elements of the fractal digital device and present them as a sum of inputs. The sum for line decoders equals the rounded product of the number of inputs by the logarithm of this number. For example, the line decoder for 13 digits has inputs equal to 13 × 5 = 65. The switching devices (SW 1.1, SW 1.2) that are triggered, depending on the value of the *n*-th bits of the MSB outputs of DC 3.2, contain 2 × 2 *(F_n_*_−1_) = 4 × *F_n_*_−1_ inputs.

The sum of inputs for *n*-bits fractal decoder:

$$S_{n}=(n-1)F_{n-1}+nF_{n-2}+4F_{n-1}=nF_{n-1}-F_{n-1}+nF_{n-2}+4F_{n-1}=nF_{n-1}+nF_{n-2}+3F_{n-1}=nF_{n}+3F_{n-1}.$$

For the line decoder device, the sum of the inputs for *n*-bits equals *n* × *F_n_*_+1_—for example, if an *n* = 5 product *n* × *F_n_*_+1_ = 5 × 13 = 65 exceeds the number of the fractal decoder elements by 10. In actual conditions, when the numbers of the code combinations of the indivisible code, as shown in Table 2, exceed ten digits, hardware costs of the fractal decoder can be significantly reduced [44].

Table 4 shows the number of fractal decoder inputs depending on *n*.

Equation (10) shows the number of inputs with minimization function *S_n_* to the number of inputs *W* = *n* × *F_n_*_+1_ fractal decoder device without minimization for *n*:

(10) $$\frac{S_{n}}{W}=\frac{nF_{n}+3F_{n-1}}{nF_{n+1}}=\frac{F_{n}}{F_{n+1}}+\frac{3F_{n-1}}{nF_{n+1}}\approx \frac{F_{n}}{F_{n+1}},$$

where *W* is the number of inputs of the fractal decoder device without minimization of the function.

The absolute value of the number of the saving hardware costs of the fractal decoder:

(11) $$\begin{array}{l} Q=nF_{n+1}-(nF_{n}+3F_{n-1})=n(F_{n}+F_{n-1})-nF_{n}-3F_{n-1}\\ =nF_{n}+nF_{n-1}-nF_{n}-3F_{n-1}=nF_{n-1}-3F_{n-1}=(n-3)F_{n-1}. \end{array}$$

Ratio *Q*/*W* determines the relative number saving hardware costs of the fractal decoder device:

(12) $$\frac{Q}{W}=\frac{nF_{n+1}-(nF_{n}+3F_{n-1})}{nF_{n+1}}=1-\frac{F_{n}}{F_{n+1}}-\frac{3F_{n-1}}{nF_{n+1}}\approx 1-\frac{F_{n}}{F_{n+1}}.$$

Equation (12) follows the *F_n_*_+1_/*F_n_* with the increase in *n* tending to the reciprocal of the golden ratio.

Table 5 shows the relative number saving hardware costs of the fractal decoder device for *n*.

For *n* = 5, the saving hardware costs of the fractal decoder device are 15.38%, and for *n* = 20, they are 34.37%, which is more than twice as high as the initial value.

**Figure 9:**
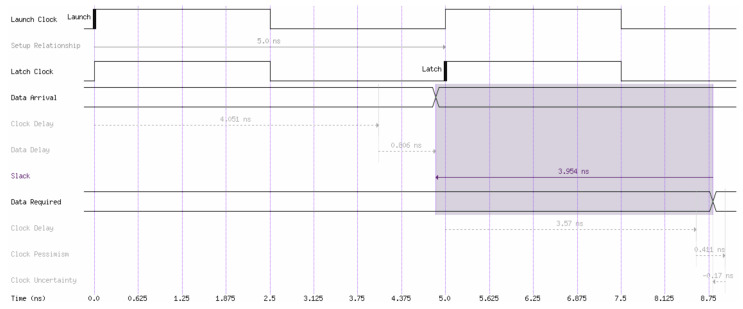
Simulation waveform of the fractal decoder device.

We realized the fractal decoder device in FPGA using Intel Quartus Prime software with the device setup of Cyclone V 5csema5f31c6. The FPGA fitting was the realization at a clock frequency of 429.37 MHz. Figure 9 shows the simulation waveform of the fractal decoder device in FPGA for *n* = 5. We utilized adaptive logic modules (ALMs) that, after modelling, were 10/32.070 and less than <1% with low power consumption. The maximal signal delay along the longest path in a combinational circuit was 0.806 ns. No digital signal processing (DSP) slices and RAM were employed. With an increase in the *n* of the fractal decoder, the delay time did not increase due to parallelization operations. The detecting ability of an indivisible code using the fractal decoder was analysed. For *n* = 64,800, the detecting errors of the indivisible code were 96%.

The fractal decoder has a block structure in which *n* increases by adding switching devices depending on the calculated number of the indivisible code. Compared to saving hardware costs, an assessment of the fractal decoder was also carried out, which reduced hardware costs from 10% to 30% compared to the implementation of line decoders based on the used codes.

## 5. Conclusions

This paper presented an FPGA-implemented fractal decoder with FEC codes in a short-reach optical interconnect model for additional control CM. An indivisible error-detecting code was evaluated on the basis of the APM. Detection of errors on average probability allowed for assessing indivisible codes with any code distance starting from *d* = 1. The fractal decoder was investigated for 56 and 35 Gbaud PAM-M (M = 4, 8) with PAM-M Gray code mapping with SSMF fibre and EDFA for interleaved BCH + LDPC and RS + LDPC codes. We used the MC method to evaluate the error statistics and post-FEC, in which interleaved concatenated RS + LDPC FEC was more efficient due to the correction of symbolic errors. Thus, it is possible to reduce the hardware costs of a fractal decoder device by using the fractal property of the fractal numbers from 10% to 30%. In some cases, with the increase in their digit capacity, this reduction was quite significant with 96% error detection. Fractal decoder devices use fewer hardware costs compared to line decoders due to the property of the fractality of the indivisible code, which provides significant advantages for the proposed device, reducing its cost, power consumption, and chip size.

## Figures and Tables

**Table 1:** Indivisible code for *n* = 8, *N* = 25, 54, 33, for bit weights 1, 2, 3, 5, 8, 13, 21, 34.

Bit Number	8	7	6	5	4	3	2	1
Bit Weight	34	21	13	8	5	3	2	1
*N* = 25	0	1	0	0	0	1	0	1
*N* = 54	1	0	1	0	1	0	1	0
*N* = 33	0	1	0	1	0	1	0	1

**Table 2:** Fractal structure of indivisible code.

	Code Combinations						Code Combinations				
	Fractal 1						Fractal 2				
**Bit Number**	**5**	**4**	**3**	**2**	**1**	**Bit Number**	**5**	**4**	**3**	**2**	**1**
**Bit Weight**	**8**	**5**	**3**	**2**	**1**	**Bit Weight**	**8**	**5**	**3**	**2**	**1**
		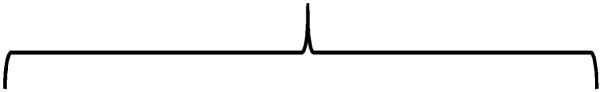						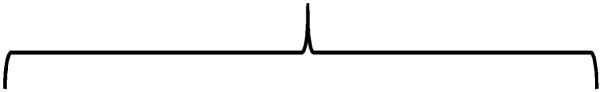			
№	x_5_	x_4_	x_3_	x_2_	x_1_	№	x_5_	x_4_	x_3_	x_2_	x_1_
0	0	0	0	0	0	8	1	0	0	0	0
1	0	0	0	0	1	9	1	0	0	0	1
2	0	0	0	1	0	10	1	0	0	1	0
3	0	0	1	0	0	11	1	0	1	0	0
4	0	0	1	0	1	12	1	0	1	0	1
		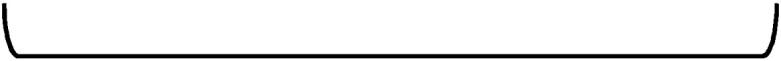						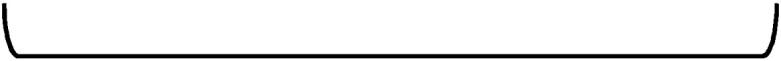			
5	0	1	0	0	0						
6	0	1	0	0	1						
7	0	1	0	1	0						

**Table 3:** Simulation setup parameters for 5 WDM optical interconnect.

Parameter Name	Value
WDM channels	5
Baud rate	56 (PAM-4), 35 (PAM-8) Gbaud
Pulse shaping	Simple
Channel frequency spacing	100 GHz
Attenuation	0.2 dB/km
Dispersion parameter	16 ps/(nm × km)
Nonlinear coefficient	2.6 × 10^−20^ m^2^/W
EDFA noise figure	3 dB
Coded modulation	PAM-M, Gray code mapping

**Table 4:** Number of fractal decoder inputs depending on *n*.

*n*	*S_n_*	*n*	*S_n_*	*n*	*S_n_*
2	7	12	3.21 × 10^3^	22	6.83 × 10^5^
3	15	13	5.59 × 10^3^	23	1.15 × 10^6^
4	29	14	9.67 × 10^3^	24	1.94 × 10^6^
5	55	15	1.66 × 10^4^	25	3.26 × 10^6^
6	102	16	2.84 × 10^4^	26	5.47 × 10^6^
7	186	17	4.87 × 10^4^	27	9.17 × 10^6^
8	335	18	8.30 ×10^4^	28	1.53 × 10^7^
9	567	19	1.41 × 10^5^	29	2.56 × 10^7^
10	1.05 × 10^3^	20	2.39 × 10^5^	30	4.28 × 10^7^
11	1.84 × 10^3^	21	4.04 × 10^5^	31	7.15 × 10^7^

**Table 5:** Relative number saving hardware costs of fractal decoder device from *n*.

*n*	*Q/W* × 100%	*n*	*Q/W* × 100%	*n*	*Q/W* × 100%	*n*	*Q/W* × 100%
5	15.38	19	32.16	12	28.77	26	33.78
6	19.23	20	32.47	13	29.17	27	33.95
7	21.76	21	32.74	14	29.88	28	34.12
8	23.89	22	32.98	15	30.58	29	34.24
9	25.45	23	33.21	16	31.05	20	34.37
10	26.74	24	33.42	17	31.42	31	34.55
11	27.97	25	33.61	18	31.81	32	34.61

## Data Availability

Not applicable.
